# Abundance and Size Distribution of the Sacoglossan *Elysia viridis* on Co-Occurring Algal Hosts on the Swedish West Coast

**DOI:** 10.1371/journal.pone.0092472

**Published:** 2014-03-19

**Authors:** Finn A. Baumgartner, Gunilla B. Toth

**Affiliations:** Department of Biological and Environmental Sciences – Tjärnö, University of Gothenburg, Strömstad, Sweden; CSIR-National Institute of Oceanography, India

## Abstract

Sacoglossans are specialized marine herbivores that tend to have a close evolutionary relationship with their macroalgal hosts, but the widely distributed species *Elysia viridis* can associate with several algal species. However, most previous investigations on the field abundance and size distribution of *E. viridis* have focussed on *Codium* spp. in the British Isles, and algae from this genus are considered superior hosts for *E. viridis*. In the present study, we investigated the abundance and size distribution of *E. viridis* on 6 potential host algae with differing morphologies (the septate species *Cladophora sericea*, *Cladophora rupestris*, *Chaetomorpha melagonium*, and *Ceramium virgatum*, as well as the siphonaceous species *Codium fragile* and *Bryopsis sp.*) at 2 sites on the Swedish west coast over the course of a year. In spring, slugs were almost absent from all algal hosts. In summer and autumn, *E. viridis* consistently occurred on several of the algal species at both sites. The highest number of small *E. viridis* were found on *C. sericea*, intermediate numbers of significantly larger *E. viridis* were found on *C. rupestris*, while fewer, intermediate sized animals were found on *C. fragile*. Throughout the study period, only a few *E. viridis* individuals were found on *C. melagonium, Bryopsis sp.*, and *C. virgatum*. Our results indicate that *E. viridis* is an annual species in Sweden, capable of exploiting co-occurring congeneric and intergeneric algal hosts with differing morphologies. These results corroborate previous findings that *E. viridis* can exploit several different algal species, but does not indicate that *C. fragile* is a superior host.

## Introduction

Sacoglossans are an intriguing clade of predominantly herbivorous opisthobranch molluscs that are considered one of the few examples of specialized herbivores in the marine environment. Each sacoglossan species is generally restricted to a single genus or species of macroalgae and tend to have a close evolutionary relationship with their algal host(s) [1]. A defining characteristic of the group is their possession of a uniseriate radula consisting of intricate modified teeth adapted to pierce different types of algal cells from which they suck cytoplasm [2]. Furthermore, many species are capable of sequestration and/or conversion of algal chemical defence compounds [3], retention of functional algal chloroplasts from their macroalgal diets, known as kleptoplasty [4], and demonstrate crypsis in both morphology and colouration [3].

Despite these adaptive traits having caught the imagination of many researchers, interpretation of their ecological relevance has been limited. This likely stems from a paucity of ecologically relevant field data on host associations in natural sacoglossan populations [5], [6] (but see [7], [8]). Observational field data are important in providing an ecological basis to test and/or explain how mechanistic studies fit within the contextual framework of natural systems [9]. Although a substantial amount of data are available on sacoglossan-algal host associations in the laboratory [6], [10], with more insight now being gained from the use of molecular techniques to accurately identify sacoglossan diets (e.g. [11]–[15]), quantitative observational field data of abundance and size distribution on algal hosts are still limited for most species (but see [5], [7], [16]–[20]).

In the present study, we focus on the widely distributed sacoglossan species *Elysia viridis*, which can be found on northeast Atlantic coasts from Norway, south, including the British Isles, into the Mediterranean Sea [21]. Unlike many sacoglossan species, *E. viridis* associates with several genera of macroalgae (e.g. [22]). However, the most commonly cited food sources for *E. viridis* are green macroalgae from the siphonaceous genera *Bryopsis* and *Codium*, and the filamentous septate genera *Cladophora* and *Chaetomorpha* [10]. *E. viridis* collected on *Codium* spp. were larger than individuals collected from *Bryopsis plumosa*, but smaller than animals collected on *Chaetomorpha* sp. [22]. Furthermore, slugs fed *Codium fragile* grew to a larger size than those fed *Cladophora rupestris*, indicating that *C. fragile* ssp. are a superior food source for *E. viridis* compared to *C. rupestris* [21].

Despite being considered one of the better-studied sacoglossan species in terms of ecology [18], [21], reproductive biology [21], [23], and kleptoplasty [24]–[26], there are surprisingly few rigorous field studies on the abundance and size distribution of *E. viridis* on different algal host species. Previous observational field abundance and size data have come from the British Isles, often focussing on macroalgae in the genus *Codium* (e.g. [18], [21], [22]). Trowbridge and Todd [21] did search for *E. viridis* on *C. rupestris* in Scottish slug populations; however, they only found *E. viridis* on *C. rupestris* when the introduced host *C. fragile* ssp. *tomentosoides* was absent. Furthermore, field populations of *E. viridis* were reported on algal hosts belonging to different genera and species including *Chaetomorpha* sp., *Cladophora* sp., *C. rupestris*, *Bryopsis* sp., and *Griffithsia* sp. [22]. However, the study by Trowbridge and co-workers [22] was not designed to statistically compare quantitative data on the abundance and size distribution of *E. viridis* on different co-occurring algal hosts.

On the west coast of Sweden the occurrence of *E. viridis* has been reported previously [27], [28], but no study on its distribution among algal hosts has, to our knowledge, been conducted in Scandinavian waters. Several genera that are potential algal hosts for *E. viridis* such as *Codium, Cladophora, Bryopsis*, and *Chaetomorpha* are fairly common and often co-occur at several sites in this region. In this study, we sampled 6 potential algal hosts during different seasons at 2 sites on the Swedish west coast in order to 1) provide data on the seasonal patterns of abundance and size distribution of *E. viridis* on algal hosts in Sweden and 2) establish if *E. viridis* commonly colonise co-occurring intergeneric and congeneric algal hosts.

## Materials and Methods

### Sampling area and algal hosts

Abundance and size of *E. viridis* on co-occurring algal hosts was assessed via 4 surveys conducted by snorkelling at 2 sites (<1–5 m depth) in the Koster Fjord, Sweden (Yttre Vattenholmen 58° 52′ 33.5″ N, 11°6′ 22.9″ E and Saltö Lyngnholmen 58° 51′ 45.3″ N, 11° 7′ 52.8″ E) from autumn 2010 to autumn 2011. No specific permissions were required to sample organisms at these locations and the study did not involve any endangered or protected species. Sites were characterized by predominantly submerged (tidal range <0.3 m, [29]) sloping to vertical rocky outcrops covered by algae.

*E. viridis* are difficult to locate directly in the field so instead of screening all potential algal hosts present at a site, the surveys focussed predominantly on 6 species. 3 green algal species, the filamentous septate species *C. rupestris* and *Cladophora sp.* (likely *sericea*/*albida* complex but will be referred to as *C. sericea* henceforth) and the siphonaceous planar *C. fragile*, were selected as they commonly co-occur at both sites in reasonable abundance during the summer and autumn months. Furthermore, a combined total of 1187 *E. viridis* were found on these hosts across both sites during a pilot survey conducted in summer 2010 (late July/early August). In addition, a co-occurring dominant red alga (*Ceramium virgatum*), which to our knowledge has not previously been reported as an *E. viridis* host, was selected to assess whether the number of slugs found on different hosts were due to the relative abundance of the algae rather than specific algal-herbivore associations. Finally, individuals of *Chaetomorpha melagonium* and *Bryopsis* sp. were collected during some surveys. *C. melagonium*, whilst present during most surveys, was not particularly abundant with individuals ranging from a single filament to several filaments often <1 g. *Bryopsis* sp. was only common/visible during the spring survey.

### Elysia viridis abundance

*E. viridis* abundance was assessed by haphazardly sampling an average of 10 samples of each potential algal host from the 2 sites (Yttre Vattenholmen and Saltö Lyngnholmen) in autumn 2010 (late October), spring 2011 (late May), summer 2011 (late July/early August), and autumn 2011 (early October). Algal individuals were carefully removed from the substrate and placed in separate press seal bags with seawater and transported back to the Sven Lovén Centre for Marine Sciences-Tjärnö to be searched for *E. viridis*. Each algal individual was thoroughly searched for *E. viridis*, the number of animals recorded, after which the alga was spun in a salad spinner, patted dry with absorbent paper in order to remove excess water, and weighed (±0.1 g). Abundance data were standardized to the number of *E. viridis* per g algal individual (no. g algal ind^−1^). Furthermore, we calculated the percentage of thalli that were colonised by *E. viridis* (% thalli occupied). This was calculated by dividing the number of thalli *E. viridis* were found on by the total number of thalli searched for each potential host species from each site in each survey and multiplying this value by 100.

### *Elysia viridis* size distribution

Size of the collected *E. viridis* individuals was measured during all surveys to assess the variation between algal hosts within sites. All *E. viridis* individuals found in the autumn collections (2010 and 2011) were patted dry on absorbent paper and weighed (±1 mg). However, in the summer collection of 2011 there were extremely high numbers of *E. viridis* (>2500 individuals) that were mostly small (<1 mg) preventing accurate assessment of their mass. Instead, *E. viridis* lengths were measured (±0.083 mm) while slugs crawled across a petri dish, using a microscope equipped with an ocular micrometer. As measuring all individuals was not practical, a subset of animals was measured to provide a relative estimate of *E. viridis* size on each algal host. A maximum of 20 individual *E. viridis* were measured from each of the first 5 algal individuals searched of each algal host from each site. This amounted to 77–100 *E. viridis* individuals measured per algal host per site for *C. sericea*, *C. rupestris* and *C. fragile*.

### Statistical analyses

As all potential algal hosts were not found at each site during each survey, we chose to analyse *E. viridis* abundance and size data from different sites and surveys in separate statistical analyses. Data from the spring survey were not statistically analysed because too few *E. viridis* were found (see Results). Data on the mean abundance (no. g algal ind.^−1^) of *E. viridis* on different algal hosts were statistically compared for each survey and site by 1-factor analysis of variance (ANOVA) with algal host as a fixed factor. Due to heterogeneous variances around the means, data on *E. viridis* size (mass, mg) at each site in the autumn surveys were statistically analysed with t-tests using separate variances [30]. Data on the size (length, mm) of *E. viridis* on different algal hosts at each site in the summer survey were compared using 1-factor ANOVAs with algal host as a fixed factor. When less than 20 *E. viridis* were found on a host alga at a particular site and survey, data were not included in the analysis in order to reduce strong bias associated with highly uneven sample sizes [30]. Data on algal host colonization (% thalli occupied by *E. viridis*) were not statistically analysed because only one value was obtained for each algal host species at each site and survey (i.e. n = 1).

Prior to all statistical analyses, data were tested for homogeneity of variances using Levene's test. All variances were heterogeneous apart from data on mean size of *E. viridis* from the summer survey, which were successfully transformed to log (x). However, since transformations did improve the homogeneity of variances, data on the size of *E. viridis* from the autumn surveys were also transformed to log (x) and the abundance data from all surveys were transformed to log (x+1). Post hoc comparisons of mean values were made using Dunnett's T3 multiple-comparisons test when variances were heterogeneous [30], or Tukey's honestly significant different (HSD) multiple-comparisons test when variances were homogeneous.

## Results

### Elysia viridis abundance

**Figure 1:**
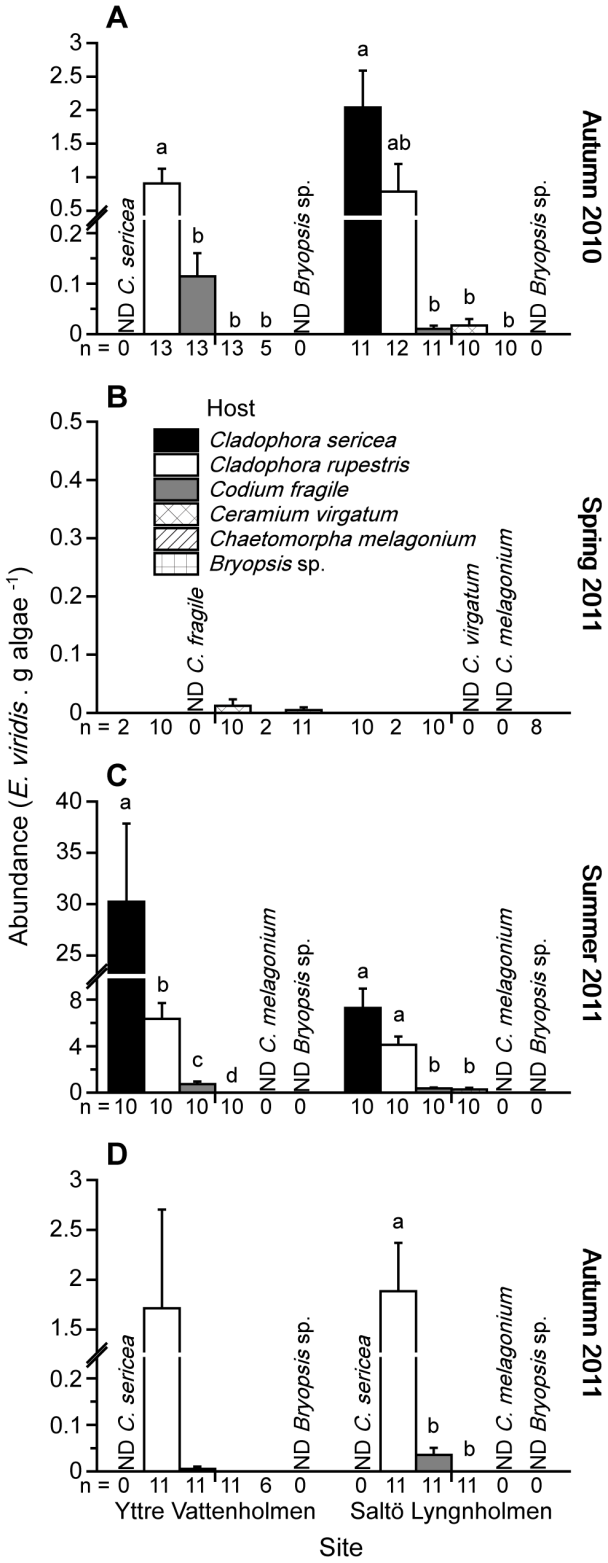
Abundance on different algal hosts. Field abundance of *Elysia viridis* (*E. viridis*. g algae^−1^; mean + SE) on different algal hosts at 2 sites (Yttre Vattenholmen and Saltö Lyngnholmen) during 4 surveys: (A) autumn 2010, (B) spring 2011, (C) summer 2011, and (D) autumn 2011. Numbers below bars indicate the number of samples of each algal host searched. ND denotes no data for a particular host, i.e. no samples of the algal host were collected. Algal hosts within a site and survey sharing the same letter do not significantly differ at α = 0.05 (Dunnett's T3 multiple comparisons test).

*E. viridis* were found on several species of co-occurring algal hosts at both sites during 3 of the surveys (autumn 2010, summer 2011, and autumn 2011; Fig. 1 A, C, D). In spring 2011, however, only 2 individuals were found combined across all algal hosts and sites searched (Fig. 1B, data not included in statistical analyses). In general, the abundance of *E. viridis* was low on the green algal species *Bryopsis* sp. and *C. melagonium*, and on the dominant red alga *C. virgatum*. In contrast, *E. viridis* numbers were substantially higher on the green algal hosts *C. sericea*, *C. rupestris* and *C. fragile* (Fig. 1). However, the number of *E. viridis* found on different algal hosts varied substantially between sites and surveys.

In autumn 2010, there was a statistically significant difference in the abundance of *E. viridis* on different algal hosts at both Yttre Vattenholmen (ANOVA, *F*_3,40_ = 13.520, p<0.001; Fig. 1A) and Saltö Lyngnholmen (ANOVA, *F*_4,49_ = 12.077, p<0.001; Fig. 1A). When means were compared using Dunnett's T3 multiple-comparisons test, we found that *E. viridis* abundance was significantly (p<0.05) higher on *C. rupestris* than on *C. fragile*, *C. virgatum*, and *C. melagonium* at Yttre Vattenholmen (Fig. 1A). *C. sericea* and *Bryopsis* sp. were not found at this site during the survey. In contrast, the abundance of *E. viridis* was significantly (p<0.05) higher on *C. sericea*, and lower on *C. fragile, C. virgatum* and *C. melagonium*, compared to *C. rupestris* at Saltö Lyngnholmen (Fig. 1A). No specimens of *Bryopsis* sp. were found at this site during autumn 2010.

In summer 2011, we found a statistically significant difference in the abundance of *E. viridis* on different algal hosts at both Yttre Vattenholmen (ANOVA, *F*_3,36_ = 70.613, p<0.001; Fig. 1C) and Saltö Lyngnholmen (ANOVA, *F*_3,36_ = 48.568, p<0.001; Fig. 1C). Post hoc comparisons (Dunnett's T3 multiple-comparisons test), revealed that *E. viridis* abundance was significantly (p<0.05) higher on *C. sericea* than on *C. rupestris*, *C. fragile* and *C. virgatum* at Yttre Vattenholmen (Fig. 1C). Furthermore, at Saltö Lyngnholmen, *E. viridis* were significantly (p<0.05) more abundant on the 2 *Cladophora* spp. than on *C. fragile* and *C. virgatum* (Fig. 1C). *C. melagonium* and *Bryopsis* sp. were not collected/found at either site during the summer 2011 survey.

Very few slugs were found at Yttre Vattenholmen in autumn 2011 (a total of 45 individuals on *C. rupestris* and 2 on *C. fragile*), and therefore data on abundance of *E. viridis* between different algal hosts were not statistically compared (Fig. 1D). *C. sericea* and *Bryopsis* sp. were not present at this site during the survey. However, there was a statistically significant difference in the abundance of *E. viridis* between different algal hosts at Saltö Lyngnholmen (ANOVA, *F*_2,30_ = 22.741, p<0.001; Fig. 1D). Dunnett's T3 multiple-comparisons test showed that *E. viridis* were significantly (p<0.05) more abundant on *C. rupestris* than on *C. fragile* and *C. virgatum* (Fig. 1D). *C. sericea*, *C. melagonium*, and *Bryopsis* sp. were not found at this site during the survey.

**Figure 2:**
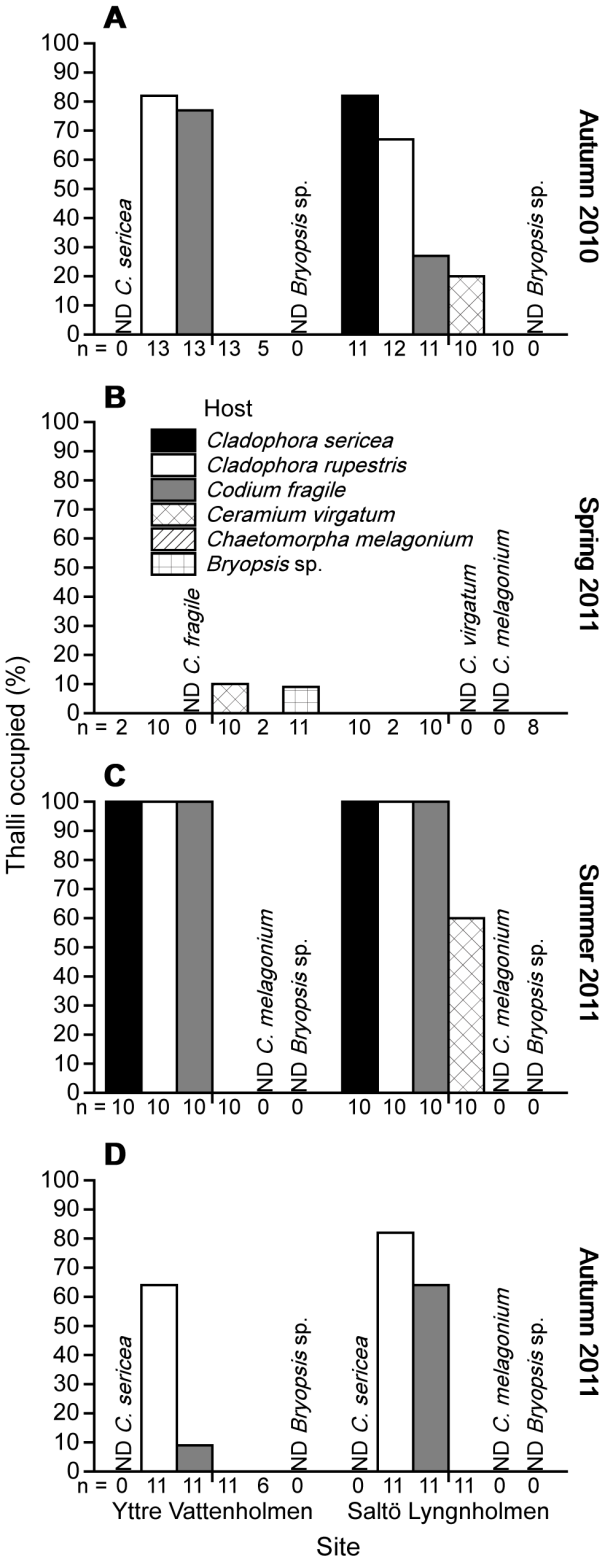
Colonisation of different algal hosts. Thalli occupied by *Elysia viridis* (%) on different algal hosts at 2 sites (Yttre Vattenholmen and Saltö Lyngnholmen) during 4 surveys: (A) autumn 2010, (B) spring 2011, (C) summer 2011, and (D) autumn 2011. Numbers below bars indicate the number of samples of each algal host searched. ND denotes no data for a particular host, i.e. no samples of the algal host were collected.

Patterns of host colonisation (i.e. the percentage of algal thalli occupied by *E. viridis*) demonstrated consistently high use of the 2 *Cladophora* hosts by *E. viridis* in summer and both autumn surveys (between 65–100% thalli occupied; Fig. 2A, C, D) compared with *C. fragile* where colonisation was more variable (between 10–100% thalli occupied; Fig. 2A, C, D). Few algal thalli were colonised by *E. viridis* in spring 2011 with only 2 individuals found, one on *Bryopsis* sp. and the other on *C. virgatum* (Fig. 2B).

### *Elysia viridis* size distribution

**Figure 3:**
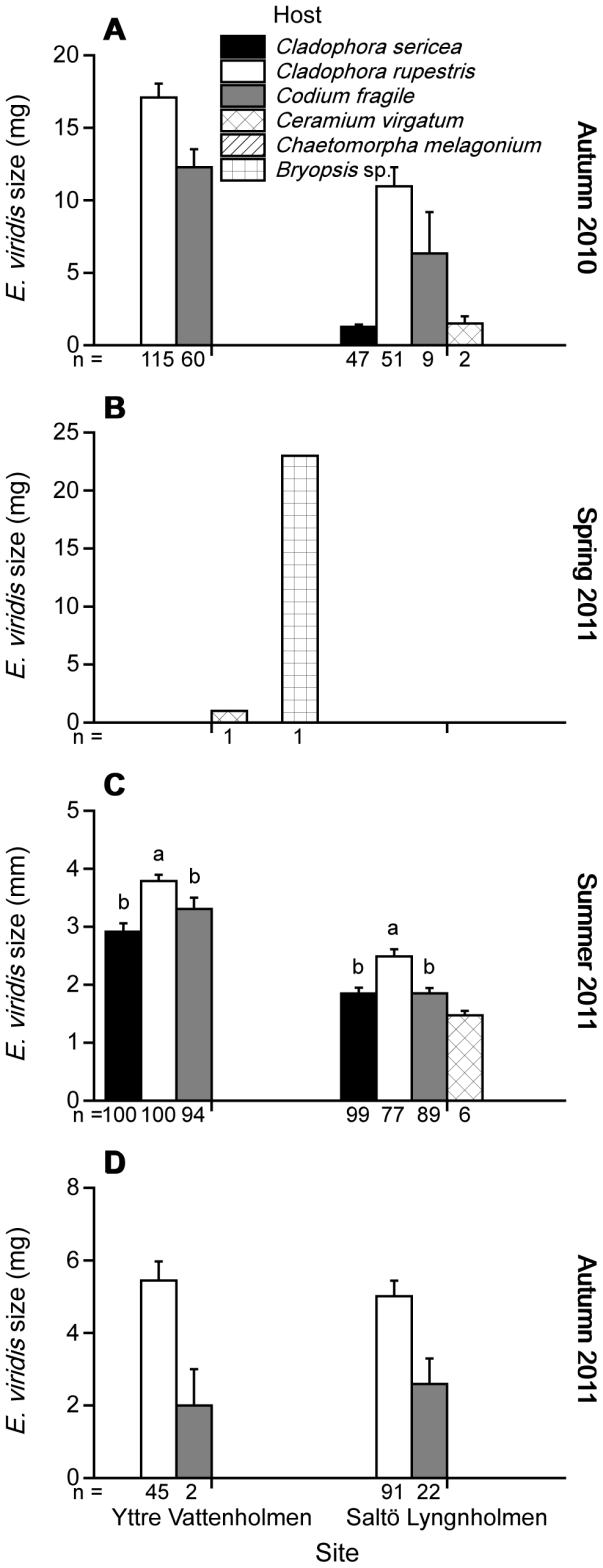
Size on different algal hosts. Size of *Elysia viridis* (mg or mm, mean + SE) collected from different algal hosts at 2 sites (Yttre Vattenholmen and Saltö Lyngnholmen) during 4 surveys: (A) autumn 2010, (B) spring 2011, (C) summer 2011, and (D) autumn 2011. Numbers below bars denote the total number of *E. viridis* measured from each algal host. Data were included in the statistical analysis when at least 20 *E. viridis* were collected from a particular host, site and survey. Algal hosts within a site and survey sharing the same letter do not significantly differ at α = 0.05 (Tukey's honestly significant difference, HSD, multiple comparisons test).

In autumn 2010, there was a statistically significant difference in the size of *E. viridis* collected from different hosts at Saltö Lyngnholmen (t-test, *t*_1,51_ = 7.30, p<0.001; Fig. 3A). *E. viridis* collected from *C. rupestris* were on average 8.6 times larger than those from *C. sericea.* Size data for *E. viridis* collected from *C. fragile* were not statistically analysed due to the low number of animals, but their mean mass lay in between animals collected from the 2 *Cladophora* spp. (Fig. 3A). At Yttre Vattenholmen, *C. sericea* was not present in autumn 2010 and this species was therefore not included in the statistical analysis. However, there was a statistically significant difference in size between *E. viridis* individuals collected from *C. rupestris* compared to those collected from *C. fragile* (t-test, *t*_1,173_ = 3.16, p = 0.003; Fig. 3A) with *E. viridis* from *C. rupestris* on average 1.4 times larger than slugs collected from *C. fragile* (Fig. 3A). Only 2 *E. viridis* individuals were found in spring 2011, 1 on *Bryopsis* sp. and the other on *C. virgatum* (Fig. 3B).

In summer 2011, there was a statistically significant difference in size between *E. viridis* collected from different algal hosts at both Yttre Vattenholmen (ANOVA, *F*_2,291_ = 7.774, p = 0.001; Fig. 3C) and Saltö Lyngnholmen (ANOVA, *F*_2,262_ = 13.904, p<0.001; Fig. 3C). However, a large proportion of the *E. viridis* individuals were in the range of 1–4 mm, and the differences in size of *E. viridis* on different hosts were small (<1 mm; Fig. 3C). *E. viridis* collected from *C. rupestris* were longer compared to individuals collected from *C. sericea* and *C. fragile* at both sites (Fig. 3C). Size data for *E. viridis* on *C. virgatum* were not included in the analysis, as only 6 individuals in total were found on this algal host.

In autumn 2011, *E. viridis* individuals collected from *C. rupestris* were significantly larger than those collected from *C. fragile* at Saltö Lyngnholmen (t-test, *t*_1,111_ = −2.58, p = 0.011; Fig. 3D). A statistical comparison could not be made between *E. viridis* collected from different hosts at Yttre Vattenholmen as only 2 *E. viridis* were found on *C. fragile* in contrast to the 45 *E. viridis* found on *C. rupestris*. However, mean *E. viridis* size on *C. rupestris* and *C. fragile* hosts were similar at both sites (Fig. 3D).

## Discussion

Sacoglossans are a group of herbivorous marine molluscs that commonly have specialized adaptations to their algal hosts and therefore also tend to have restricted taxonomic host associations [1]. However, some sacoglossans, e.g. several species in the genus *Elysia*, have less restricted host use, associating with/feeding on algae from several different families (e.g. [12], [14], [16], [20], [31]). We surveyed the abundance and size of *E. viridis* on 6 different potential host algae (the green algal species *C. sericea, C. rupestris, C. fragile, C. melagonium, Bryopsis* sp., and the red alga *C. virgatum*) at 2 sites on the Swedish west coast. To our knowledge, this is the first investigation that has rigorously tested the abundance and size distribution of *E. viridis* on several co-occurring macroalgal hosts through statistical comparisons of data acquired through structurally designed collections. In spring, slugs were almost absent from all algal hosts. In summer and autumn, *E. viridis* consistently occurred on several of the algal species at both sites. The highest number of small *E. viridis* were found on *C. sericea*, intermediate numbers of significantly larger *E. viridis* were found on *C. rupestris*, while fewer, intermediate sized animals were found on *C. fragile*. Throughout the study period, few *E. viridis* individuals were found on *C. melagonium, Bryopsis sp.*, and *C. virgatum*.

Previous investigations of *E. viridis* abundance and size distribution on different algal hosts have been conducted in the British Isles (e.g. [18], [21], [22], [32]). These studies were primarily designed to study the interaction between *E. viridis* and the introduced species *C. fragile* spp. *tomentosoides* and did not sample several co-occurring potential algal host species simultaneously (but see [21] where the authors searched for *E. viridis* on *C. fragile* and *C. rupestris* simultaneously) nor did they report quantitative, statistically analysed abundance and size data of *E. viridis* on the other algal host species. However, *E. viridis* were found to occur on *Codium* spp., *Cladophora* spp., and *Chaetomorpha* sp. within a 1 km^2^ Irish lough, as well as on *Codium* spp., *Chaetomorpha* sp., and *Bryopsis* sp. at other sites in the British Isles [18], [22], indicating a broad taxonomic host use. Scottish populations of *E. viridis*, on the other hand, demonstrated a more selective pattern of host use in which sacoglossans only occurred on *C. rupestris* when *C. fragile spp. tomentosoides* was absent [21]. In our study, few *E. viridis* were found on *C. melagonium* and *Bryopsis* sp., probably because the biomass of these species was low (*C. melagonium* often grew as single filaments and *Bryopsis* sp. was only present in spring when *E. viridis* was mostly absent). Furthermore, *E. viridis* colonised *C. rupestris* hosts at percentages that were either the same or higher compared to *C. fragile*, and *E. viridis* were larger and more abundant on *C. rupestris*, indicating that *C. rupestris* is a better host than *C. fragile* on the Swedish west coast.

Apart from a broad taxonomic host use, we found an annual variation in the abundance and size of *E. viridis* on the Swedish west coast. *E. viridis* were mostly absent in the spring survey, while high numbers of small slugs were found in summer. In autumn, *E. viridis* were larger but lower in number. In contrast, [21] consistently observed large *E. viridis* throughout the winter, spring, and summer in Scotland. Results from the present study indicate that *E. viridis* is an annual species in Sweden, and that the larvae that settle in spring or early summer may arise from external populations. *E. viridis* larvae have a large potential for dispersal (*E. viridis* larvae are planktonic for *ca.* 30 days, [23]), and it might be hypothesized that long distance transport, and hence increasing age prior to settlement, make the larvae “desperate” (i.e. settling on any host), thereby reducing host selectivity and resulting in a broader host-use pattern. However, *E. viridis* did not demonstrate a pattern suggesting desperate larvae evidenced by their consistently low abundance/absence on *C. virgatum* compared to the less common green algal hosts in summer and autumn surveys.

Host selection in natural populations of *E. viridis* may be controlled by physical and/or biological constraints (e.g. removal via wave motion, and/or predation), although their impacts on *E. viridis* distributions are not yet well understood [33]. Increased intensity of wave exposure has been linked to changes in size distribution and decreased abundance of the sacoglossan *Placida dendritica* [34], [35], and algal host complexity has been suggested to increase the abiotic refuge value for amphipods [36]. Physical disturbance through wave motion may explain the general decreases in *E. viridis* abundance observed from summer to autumn surveys in the present study, as strong autumn storms are common in the Koster Fjord and may facilitate variable removal of *E. viridis* from different hosts. Furthermore, although we are not aware of any published reports of predators that use *E. viridis* as prey, several potential predators (e.g. different species of crabs and fish) co-occur with *E. viridis* in the study area (G. B. Toth, pers. obs.). Therefore, predation could also be a potential, but not mutually exclusive, explanation for the seasonal decreases in *E. viridis* abundance and variation in size structure between hosts from summer to autumn. Algal host morphology and colour could contribute to the probability of *E. viridis* survival. Small *E. viridis* were often visible on the light green *C. sericea* whilst snorkelling (F. Baumgartner, pers. obs.) and although anecdotal, this evidence might suggest detection by visual predators could be substantial, explaining the lack of any large *E. viridis* on this alga. Furthermore, the morphology of *C. fragile* and *C. melagonium* likely provides little refuge through crypsis when *E. viridis* are large.

The broad taxonomic host-use pattern observed in our study area and in the British Isles could be due to ontogenetic or adult host switching by *E. viridis*, or to differential host exploitation by different *E. viridis* individuals within populations. *E. viridis* demonstrates variation in feeding behaviour at several ontogenetic stages (e.g. [21], [22], [37]), but initial genetic studies have revealed no apparent differences between *E. viridis* associated with different algal hosts [22]. Together, these results indicate that *E. viridis* is capable of host switching in some areas. Host selection, intra- and inter- specific host specialization, and host switching are considered important mechanisms for sacoglossan speciation [1]. For example, speciation within the genus *Elysia* has been linked to algal host switches with sympatric congeners commonly exploiting different algal diets [1]. Furthermore intergeneric and congeneric algal host use by sympatric conspecific sacoglossans has been observed ([8]: *Aplysiopsis enteromorphae*, [17]: *Placida dendritica*, [20]: *Elysia clarki*, [22]: *E. viridis*) and, although considered rare in this clade, intraspecific variation and/or specialization in host use are hypothesized to be important sources of polymorphism and processes for speciation in many other groups of animals [38].

In conclusion, host selection in small, sedentary herbivores like *E. viridis*, which use algal individuals both as food and shelter, most likely depends on a combination of several intrinsic algal and herbivore traits, as well as extrinsic physical and biological constraints. From our data it is clear that *E. viridis* colonise co-occurring congeneric and intergeneric green algal hosts on the west coast of Sweden with *C. rupestris* seemingly providing the most stable host. This quantitative observational data will be vital in providing a framework to which mechanistic studies on intraspecific variation in host use can be related. Furthermore, it will provide a stronger ecological basis to address questions regarding both physical and biological constraints on *E. viridis* abundance and size distributions. The fact that so few studies have focussed on the distribution of *E. viridis* in natural field populations is surprising considering that several aspects of *E. viridis* biology and behaviour have been well-studied in the laboratory (e.g. [21], [23]–[26]). To be able to draw ecologically relevant conclusions about the natural history, biology and/or ecology of a species, results from laboratory experiments must be compared with observational field data.
